# Transcriptome analysis of drought-tolerant sorghum genotype SC56 in response to water stress reveals an oxidative stress defense strategy

**DOI:** 10.1007/s11033-020-05396-5

**Published:** 2020-04-17

**Authors:** Farida Azzouz-Olden, Arthur G. Hunt, Randy Dinkins

**Affiliations:** 1 Kentucky State University, 400 East Main Street, Frankfort, KY 40601 USA; 2 Department of Plant and Soil Sciences, University of Kentucky, Lexington, KY 40546 USA; 3 USDA-ARS Forage-Animal Production Research Unit, 1100 Limestone Rd, Lexington, KY 40546 USA

**Keywords:** RNA-seq, Drought, Stress, Tolerance, Sorghum, Stay-green

## Abstract

Drought tolerance is a crucial trait for crops to curtail the yield loss inflicted by water stress, yet genetic improvement efforts are challenged by the complexity of this character. The adaptation of sorghum to abiotic stress, its genotypic variability, and relatively small genome make this species well-suited to dissect the molecular basis of drought tolerance. The use of differential transcriptome analysis provides a snapshot of the bioprocesses underlying drought response as well as genes that might be determinants of the drought tolerance trait. RNA sequencing data were analyzed via gene ontology enrichment to compare the transcriptome profiles of two sorghum lines, the drought-tolerant SC56 and the drought-sensitive Tx7000. SC56 outperformed Tx7000 in wet conditions by upregulating processes driving growth and guaranteeing homeostasis. The drought tolerance of SC56 seems to be an intrinsic trait occurring through overexpressing stress tolerance genes in wet conditions, notably genes acting in defense against oxidative stress (SOD1, SOD2, VTC1, MDAR1, MSRB2, and ABC1K1). Similarly to wet conditions, under drought, SC56 enhanced its transmembrane transport and maintained growth-promoting mechanisms. Under drought, SC56 also upregulated stress tolerance genes that heighten the antioxidant capacity (SOD1, RCI3, VTE1, UCP1, FD1, and FD2), regulatory factors (CIPK1 and CRK7), and repressors of premature senescence (SAUL1). The differential expression analysis uncovered biological processes which upregulation enables SC56 to be a better accumulator of biomass and connects the drought tolerance trait to key stress tolerance genes, making this genotype a judicious choice for isolation of tolerance genes.

**Electronic supplementary material:**

The online version of this article (10.1007/s11033-020-05396-5) contains supplementary material, which is available to authorized users.

## Introduction

Drought is the abiotic stress that is the most devastating to crop productivity and the most recalcitrant to classical plant improvement strategies. In plants, water acts as a solvent, a transport medium, and an evaporative coolant. Thus, water limitation causes a decrease in plant growth and photosynthesis, wilting, stomatal closure, and is associated with changes in nitrogen metabolism [1]. Drought tolerance is the ability of a plant to maintain physiological activities, when tissue water potential is low, through the regulation of metabolic pathways that reduce or repair the stress damage. One of the most efficient mechanisms of drought tolerance is osmotic adjustment by which plants accumulate compatible solutes such as sugars, amino acids, or ions that lower the osmotic potential and maintain turgor in shoots and roots [2]. Another mechanism is the detoxification of reactive oxygen species (ROS) that cause oxidative stress which results in cell injury [3]. To prevent this damage, plants have evolved antioxidant pathways that involve enzymes such as superoxide dismutases, catalases, and peroxidases, as well as non-enzymatic pathways relaying on ROS scavengers such as carotenoids, ascorbic acid, proline, and tocopherols.

Drought tolerance in *Sorghum bicolor (L.) Moench* is consistent with its evolution in an African region characterized by harsh climatic conditions with poor, droughty, and infertile soils. Drought adaptation in sorghum relies on a C4 photosynthesis mechanism that enables increased net carbon assimilation under water deprivation and makes this crop one of the most efficient biomass accumulators [4]. In addition to the overall greater drought resistance of sorghum compared to other crops, certain sorghum genotypes that are more tolerant to drought than others exhibit a stay-green character that expresses post-anthesis and enables the continuation of photosynthesis and grain filling in dry conditions. These traits and the availability of its genome sequence have put sorghum in the forefront as a model system to elucidate the mechanisms of environmental stress tolerance, especially the response to drought [5, 6].

The genetic basis of adaptation to adverse environments is complex, which is consistent with the large number of developmental, biochemical, and physiological responses plants deploy in response to constraints. Often, other overlapping stresses further complicate drought’s impact on growth and metabolism, adding more challenges in selecting for this character. The dissection of the molecular response to drought has uncovered a complex hierarchy of regulatory networks modulating dehydration-induced effectors [7]. The elucidation of these networks allows the identification of key players of drought tolerance that can be validated through transgenic overexpression or knockdown studies. In the case of sorghum, despite its importance as a model crop for dissecting drought tolerance, few candidate genes conferring this trait have been identified. This reflects an ongoing need for the characterization of sorghum genes. In fact, approximately, half of the protein coding genes in sorghum have not been validated experimentally and 14% have unknown protein functions [4] leading to recent annotation efforts for discovery of drought tolerance genes [8]. In the present study, we undertook a comparative transcriptome analysis of two sorghum genotypes contrasting in their tolerance to post-anthesis drought stress: the stay-green, drought-tolerant SC56 and the drought-sensitive Tx7000 [9]. The comparison included wet conditions and post-anthesis drought to uncover the subtle differences in gene expression between both genotypes and identify drought tolerance genes, including those constitutively induced, that might be of value in plant improvement programs.

## Materials and methods

### Drought and water treatment trials

The seeds of SC56 and Tx7000 were obtained from the Plant Genetic Resources Conservation Unit of USDA-ARS, Griffin, Georgia. The trials for transcriptome profiling under drought (treated) and water (control) conditions were conducted in the greenhouse at the USDA-ARS on the University of Kentucky campus in Lexington, KY. The experiment was conducted in three biological replicates (each containing ten potted plants constituting technical replicates) for four treatment groups that comprised, SC56 drought treatment (S_treat), SC56 watered (S_cont), Tx7000 drought treatment (Tx_treat) and Tx7000 watered (Tx_cont). The seeds were treated with 1% sodium hypochlorite, thoroughly washed, soaked overnight in distilled water, and transferred to moist germination paper for 3 days. The germinated seeds were then planted in pots filled with a sand/soil mixture. Five seeds were sown per pot and thinned to one plant when stands became established. Greenhouse controls were set at daytime and nighttime temperatures of 31 °C and 22 °C, respectively, with a 12-h photoperiod. Pots were irrigated daily to the maximum of substrate capacity until the onset of anthesis. Then, watering was sustained for half of the plants that constitute the controls and withheld for the other half that constitute the water stress treatments. The soil moisture content (SMC) was monitored using a Delta-T theta probe. At 13 days post-drought, when the SMC had reached less than 10% for several days already, which is considered severe stress for sorghum, leaf material was collected for the subsequent transcriptome analysis. For each biological replicate, ten plants were randomly chosen, leaf tissue was harvested, flash frozen in liquid nitrogen, and stored at -80 °C until used for RNA extraction.

### RNA extraction and library preparation

For each biological replicate, leaf tissue samples of ten randomly chosen plants were subjected to RNA extraction using the RNeasy Plant Mini Kit (Cat No./ID: 74,904, Qiagen; Germantown, MD). The resulting ten RNA extracts were combined in equimolar quantities to form an RNA pool representing a single sample of the corresponding biological replicate. Thus, a total of 30 plants were sampled for each treatment and grouped into three RNA pools, which were used to produce sequencing libraries following a protocol described elsewhere [10].

### Analysis of sequenced data

Illumina platform HiSeq (Illumina, Inc.; San Diego, CA) was used to perform next-generation sequencing (NGS). Subsequent analysis was completed using CLC Genomics Workbench 7.5.1 (Qiagen, Bioinformatics; Redwood City, CA, USA). First, raw data were preprocessed for duplicate removal and demultiplexed to separate libraries. The ‘RNA-seq Analysis’ toolbox was utilized for sequencing mapping against the reference sorghum genome bicolor_255_V2.0 available on Phytozome 12 (https://phytozome.jgi.doe.gov/pz/portal.html). Mapping options were set at mismatch cost 2, insertion cost 3, depletion cost 3, length fraction 0.5, similarity fraction 0.8, and gene expression value set to RPKM. The mapping step resulted in all reads being assigned to specific genes (when possible) with corresponding expression value. Differential expression analysis was performed with the ‘transcriptomics analysis’ toolbox of the CLC Bio workbench. It comprised ‘experiment set-up’, where treatments pairs were analyzed with the option ‘All group pairs’. The analyzed pairs were as follows: S_treat vs. S_cont, Tx_treat vs. Tx_cont, S_treat vs. Tx_treat, and S_cont vs. Tx_cont. The ‘all group pairs’ setting, which uses Wald test, was utilized to estimate the expression mean of each gene as well as its fold change between the considered treatment pair. Expression values were normalized using the options ‘by totals’ and ‘state numbers in read 1,000,000′. The normalized values were transformed using ‘Add a Constant’ set at the value ‘1′. To identify the differentially expressed genes (DEGs) between a pair of treatments, a t-test was performed on the transformed values for each mapped gene, and DEGs were filtered based on an expression level of a two-fold change and a p-value < 0.05. To draw biological meaning, gene ontology (GO) analysis was conducted using the agriGO web-based GO toolkit and database (https://bioinfo.cau.edu.cn/agriGO/analysis.php).

### Identification of annotated drought tolerance genes

The *Arabidopsis thaliana* orthologs of the sorghum DEGs of this study were identified using the sorghum transcriptome database (https://sorghum.riken.jp/morokoshi/search.cgi?). *Arabidopsis thaliana* drought-responsive gene list (Additional file 1: Table S1) was downloaded from Phytozome using the ‘Tools’ option with the key words ‘drought stress tolerance’. The DEGs lists for S_treat vs. Tx_treat, S_cont vs. Tx_cont, S_treat vs. S_cont, and Tx_treat vs. Tx_cont were compared to the list of *Arabidopsis* drought-responsive genes, in order to mine potential drought tolerance genes. The bulk of DEGs identified by the comparisons constituted the comprehensive list of known drought-responsive genes in the present study.

## Results

### Deep sequencing and differential expression analysis

**Fig. 1:**
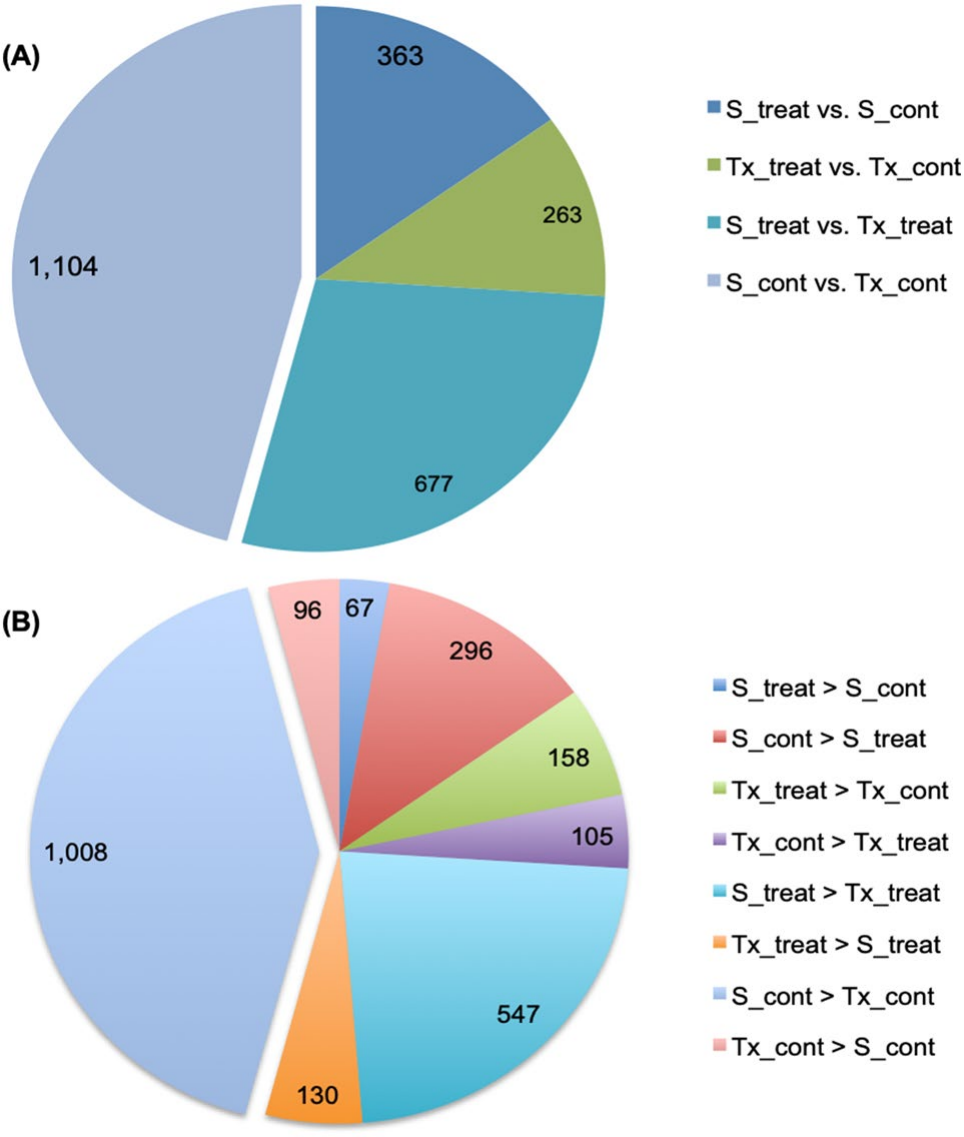
Pie chart of the number of differentially expressed genes. **a** Sections depict the experimental groups comparisons tagged with their respective total number of DEGs. **b** Sections represent group comparisons tagged with their corresponding number of upregulated DEGs. (Color figure online)

**Fig. 2:**
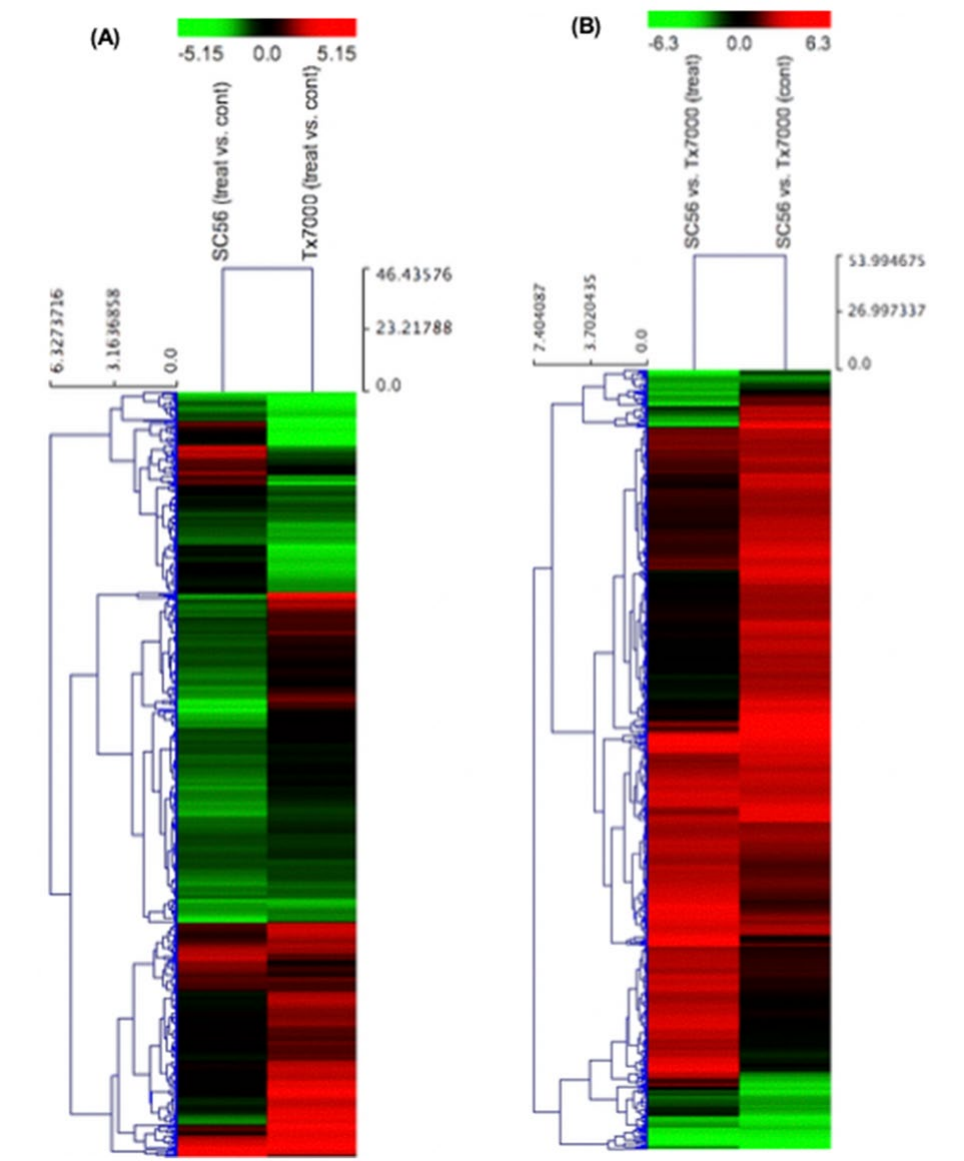
Genotype and water effects on gene regulation. **a** Genes differentially regulated in SC56 [SC56 (treat vs. cont)] and in Tx7000 [Tx7000 (treat vs. cont)] due to water regimen effect. b Genes differentially regulated due to genotype effect under drought [SC56 vs. TX7000 (treat)] and under irrigation [SC56 vs. TX7000 (cont)]. (Color figure online)

The trials for transcriptome profiling under drought (treated) and water (control) conditions comprised three biological replicates for four treatment groups: SC56 drought treatment (S_treat), SC56 watered (S_cont), Tx7000 drought treatment (Tx_treat) and Tx7000 watered (Tx_cont). Hereafter, the control conditions will be referred to as irrigated, wet or water conditions. The mapping statistics of the RNA-seq analysis are summarized in Table 1. The expression analysis of S_treat vs. Tx_treat, S_cont vs. Tx_cont, S_treat vs. S_cont, and Tx_treat vs. Tx_cont generated a total of 2,407 DEGs. The partition of this number, regardless of direction of regulation and according to the experimental factors ‘water supply’ and ‘genotype’, is detailed in Fig. 1a. Briefly, the overall responsiveness to differential water conditions (treat vs. cont) was more pronounced in the drought-resistant SC56 than in the sensitive genotype Tx7000, since 100 more DEGs were seen in the S_treat vs. S_cont comparison than the Tx_treat vs. Tx_cont comparison. The further partition of DEGs count according to the direction of regulation revealed a strong demarcation in the transcriptional tendencies of the drought-tolerant SC56 and the drought-sensitive Tx7000 lines (Figs. 1b, 2). SC56 upregulated more genes under control conditions than drought, while Tx7000 upregulated more genes under drought than under control conditions (Fig. 1b, 2a). Under wet conditions (cont vs. cont), SC56 upregulated more than ten times the number of genes overexpressed in Tx700 (Figs. 1b, 2b). Similarly, under drought conditions (treat vs. treat), SC56 upregulated more than four times the number of genes overexpressed in Tx7000 (Figs. 1b, 2b).

**Table 1:** Summary of RNA-seq mapping statistics

	Count	Percentage of reads	Average length	Number of bases
References	1610	–	451,314.66	726,616,606
Mapped reads	63,310,796	82.41%	100.59	6,368,251,025
Not mapped reads	13,517,848	17.59%	100.29	1,355,644,180
Total reads	76,828,644	100.00%	100.53	7,723,895,205

**Fig. 3:**
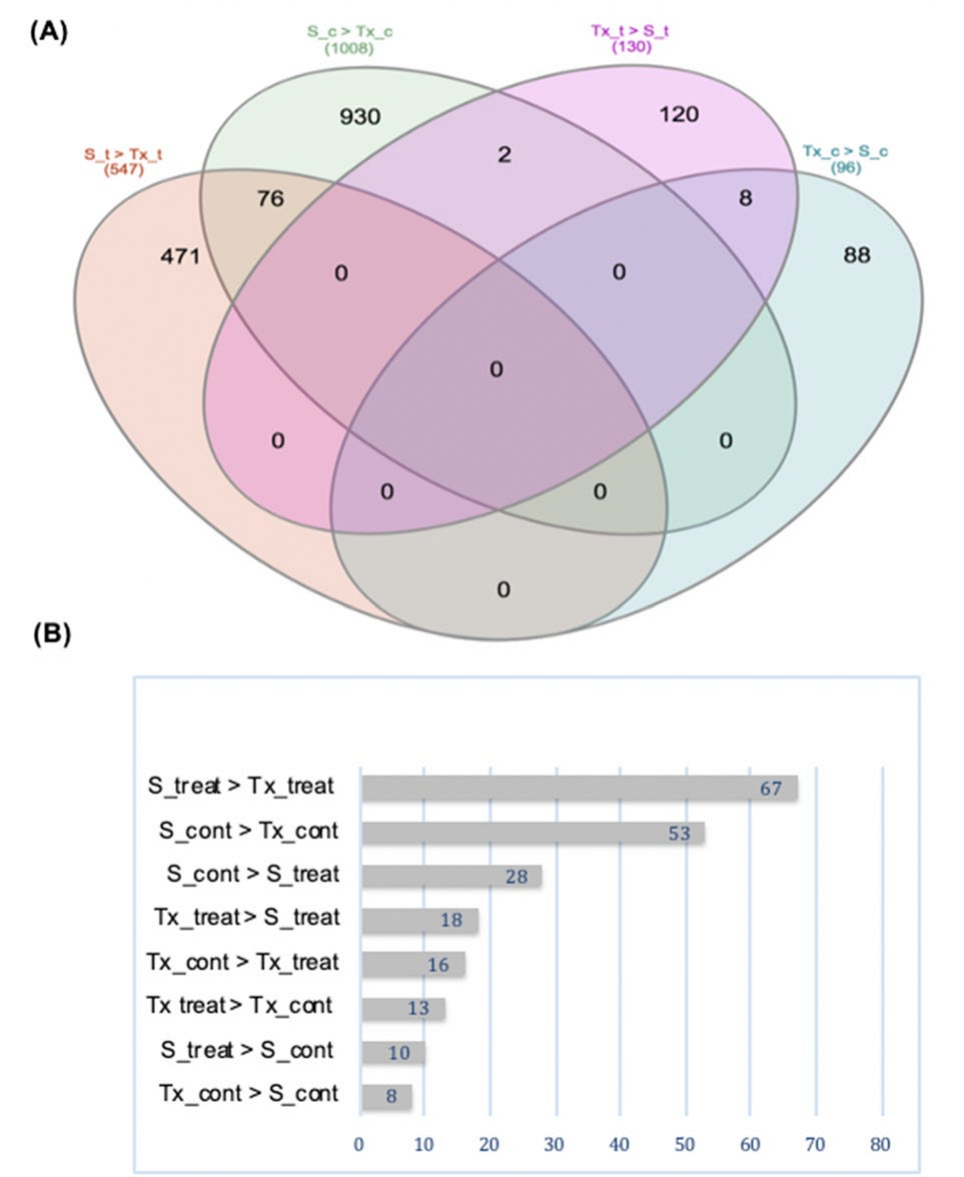
Numbers of upregulated DEGs by experimental group. **a** The Venn-diagram shows the number of non-overlapping DEGs (tags in non-common areas) as well as the number of overlapping transcripts between groups (intersecting areas). The depicted comparisons are the upregulation by SC56 versus Tx7000 under drought (S_t > Tx_t) and irrigation (S_c > Tx_c), and the upregulation by Tx7000 versus SC56 under drought (Tx_t > S_t) and irrigation (Tx_c > S_c). **b** Number of upregulated DEGs overlapping with the Arabidopsis list of drought responsive list from Phytozome. The considered comparisons are upregulation by drought treated SC56 compared to its control and treated Tx7000 (respectively, S_treat > S_cont and S_treat > Tx_treat), drought treated Tx7000 compared to its control and treated SC56 (respectively, Tx_ treat > Tx_cont and Tx_treat > S_treat), controls of both genotypes (S_cont > Tx_cont and Tx_cont > S_cont), and control versus treated (S_cont > S_treat and Tx_cont > S_treat). (Color figure online)

**Fig. 4:**
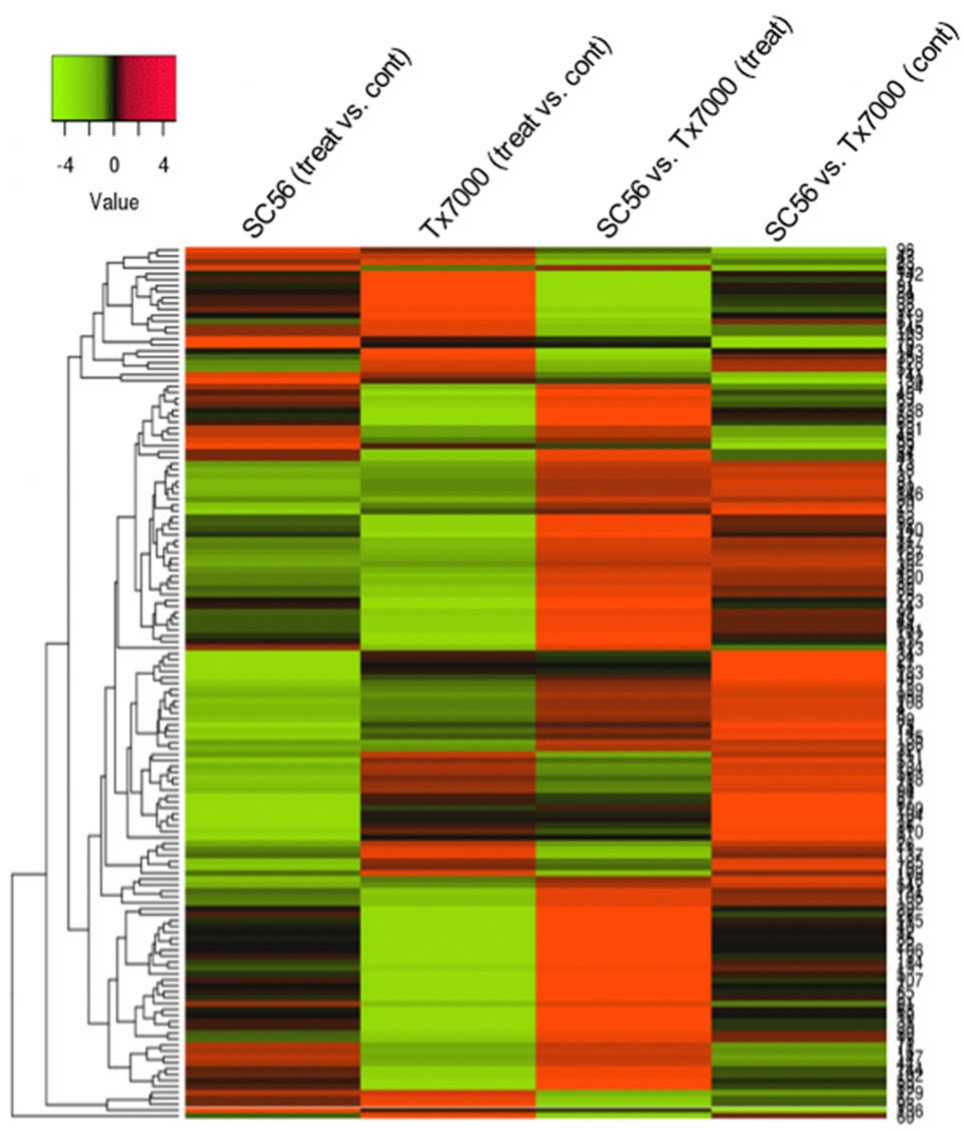
Hierarchical clustering of known drought-responsive genes identified in the differential expression analysis. Significant DEGs (p value < 0.05), at least in one comparison, are represented. Reported *A. thaliana* drought response orthologs were identified by mining the Phytozome database, and used to examine the DEG lists of the present study. Heatmap generated with parameters set to Euclidian distance and average linkage clustering method. (Color figure online)

The examination of the upregulated genes overlap between the experimental groups (Fig. 3a) uncovered 8 DEGs in common in Tx_treat > S_treat and Tx_cont > S_cont. The greatest overlap however, which counts 76 DEGs, was between S_treat > Tx_treat and S_cont > Tx_cont and is particularly important for discovery of drought tolerance genes in SC56 (see “Discussion” section). Close scrutiny of the bulk of DEGs identified in this study against a list of known drought-responsive genes in *Arabidopsis* (Additional File 1: Table S1) uncovered a total of 148 transcripts (Additional File 1: Table S3), with the largest number originating from the upregulation in SC56 compared to Tx7000 under drought conditions, while fewer genes were regulated within each genotype under differential water status (Fig. 3, and Additional File 1: Table: S2). The general regulation patterns of these genes showed a clear tendency toward upregulation in SC56 compared to Tx7000, not only in drought conditions but also in wet conditions (Fig. 4). When compared to its respective control, each genotype triggered fewer genes (Fig. 3a, b), including fewer known drought-responsive genes (Fig. 4).

### Gene Ontology Analysis

#### Effect of irrigation on gene upregulation

**Fig. 5:**
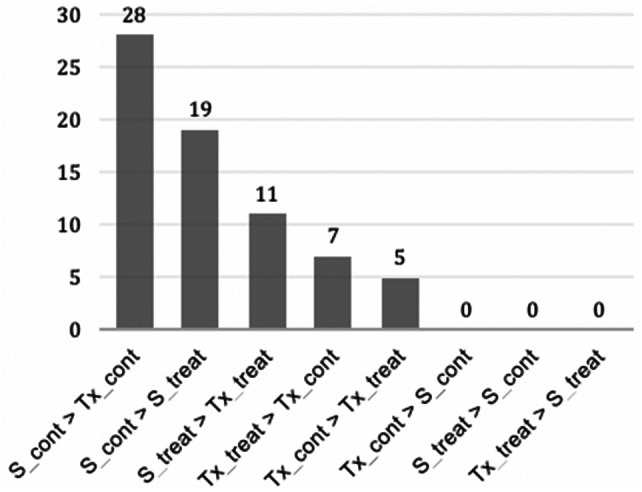
Number of upregulated biological processes in SC56 and Tx7000. Considered comparisons: upregulation by drought treated SC56 compared to its control and treated Tx7000 (respectively, S_treat > S_cont and S_treat > Tx_treat), drought treated Tx7000 compared to its control and treated SC56 (respectively, Tx_ treat > Tx_cont and Tx_treat > S_treat), controls of both genotypes (S_cont > Tx_cont and Tx_cont > S_cont), and control versus treated (S_cont > S_treat and Tx_cont > S_treat

Irrigation influenced the metabolism and biosynthesis of both genotypes, but SC56 response to irrigation (S_cont > S_treat) was more pronounced than the sensitive genotype (Tx_cont > Tx_treat) as indicated by the number of upregulated bioprocesses (Additional File 2 and Fig. 5) and the number of genes enriching these functions (Additional File 2 and Table 2). Compared to drought, irrigation of SC56 prompted the upregulation of a number of bioprocesses consistent with biosynthesis and homeostasis (Table 2). These include ‘translation’, ‘biosynthetic process,’ ‘cellular homeostasis’ and ‘regulation of biological quality’. Similarly, compared to drought, irrigation of Tx7000 prompted the upregulation of biological processes congruent with biosynthesis and heightened metabolism such as ‘translation’ and ‘macromolecule metabolic process’ (Table 2).

**Table 2:** Upregulated GO terms under water conditions compared to drought

	GO term	Term	# Genes	P value
**SC56_cont > SC56_ treat**	GO:0,006,412	Translation	16	2.10E − 05
	GO:0,044,237	cellular metabolic process	73	0.00053
	GO:0,010,467	Gene expression	32	0.0012
	GO:0,009,987	Cellular process	87	0.0014
	GO:0,009,058	Biosynthetic process	42	0.0016
	GO:0,044,249	Cellular biosynthetic process	38	0.0022
	GO:0,008,152	Metabolic process	104	0.0032
	GO:0,006,457	Protein folding	5	0.0069
	GO:0,006,807	Nitrogen compound metabolic process	33	0.0082
	GO:0,045,454	Cell redox homeostasis	5	0.01
	GO:0,019,725	Cellular homeostasis	5	0.011
	GO:0,042,592	Homeostatic process	5	0.012
	GO:0,034,645	Cellular macromolecule biosynthetic process	29	0.017
	GO:0,009,059	Macromolecule biosynthetic process	29	0.018
	GO:0,034,641	Cellular nitrogen compound metabolic process	8	0.019
	GO:0,065,008	Regulation of biological quality	5	0.024
	GO:0,044,260	Cellular macromolecule metabolic process	52	0.043
	GO:0,044,238	Primary metabolic process	72	0.044
	GO:0,006,139	Nucleobase, nucleoside, nucleotide and nucleic acid metabolic process	26	0.058
**Tx7000_cont > Tx7000_treat**	GO:0,006,412	Translation	6	0.0026
	GO:0,019,538	Protein metabolic process	15	0.018
	GO:0,010,467	Gene expression	10	0.044
	GO:0,044,267	Cellular protein metabolic process	12	0.045
	GO:0,043,170	Macromolecule metabolic process	20	0.051

##### Drought effect on upregulation

In response to water deprivation, and compared to the water conditions, SC56 did not show any enriched biological process that was significantly upregulated (Fig. 5). In contrast, drought had an upregulatory effect on Tx7000 genotype since it prompted the overexpression of certain biological processes related to signaling and metabolism (Additional file 2 and Table 3).

**Table 3:** Drought effect on upregulation of biological processes in Tx7000

GO term	Biological process	# Genes	P value
GO:0,007,165	Signal transduction	6	0.0029
GO:0,046,483	Heterocycle metabolic process	5	0.0045
GO:0,023,046	Signaling process	6	0.0071
GO:0,023,060	Signal transmission	6	0.0071
GO:0,023,052	Signaling	7	0.011
GO:0,044,262	Cellular carbohydrate metabolic process	6	0.027
GO:0,034,641	Cellular nitrogen compound metabolic process	5	0.039

##### Genotype effect on gene upregulation under wet conditions

The GO analysis revealed that under water conditions the drought-sensitive Tx7000 showed no significantly enriched biological processes that were upregulated compared to the drought-resistant genotype, SC56. In contrast, under the same conditions, SC56 upregulated the biosynthetic activity of several important cellular compounds comparted to Tx7000. Additionally, among the biological processes differentiating SC56 from Tx7000 are homeostasis-type processes as well as several metabolic processes (Additional file 2 and Table 4).

**Table 4:** Upregulated biological processes in SC56 compared toTx7000 under water conditions

GO term	Term	# Genes	p value
GO:0,006,412	Translation	31	0.00016
GO:0,044,106	Cellular amine metabolic process	17	0.0016
GO:0,034,641	Cellular nitrogen compound metabolic process	22	0.0016
GO:0,006,520	Cellular amino acid metabolic process	16	0.0026
GO:0,006,519	Cellular amino acid and derivative metabolic process	17	0.0032
GO:0,009,308	Amine metabolic process	18	0.0034
GO:0,019,725	Cellular homeostasis	11	0.0044
GO:0,042,592	Homeostatic process	11	0.0053
GO:0,009,312	Oligosaccharide biosynthetic process	5	0.008
GO:0,045,454	Cell redox homeostasis	10	0.011
GO:0,051,336	Regulation of hydrolase activity	5	0.012
GO:0,065,008	Regulation of biological quality	11	0.018
GO:0,009,058	Biosynthetic process	105	0.018
GO:0,006,399	tRNA metabolic process	9	0.018
GO:0,043,038	Amino acid activation	6	0.02
GO:0,043,039	tRNA aminoacylation	6	0.02
GO:0,009,311	Oligosaccharide metabolic process	5	0.022
GO:0,009,966	Regulation of signal transduction	5	0.026
GO:0,023,051	Regulation of signaling process	5	0.026
GO:0,010,646	Regulation of cell communication	5	0.026
GO:0,034,660	ncRNA metabolic process	10	0.027
GO:0,044,281	Small molecule metabolic process	35	0.028
GO:0,043,436	Oxoacid metabolic process	20	0.031
GO:0,019,752	Carboxylic acid metabolic process	20	0.031
GO:0,006,082	Organic acid metabolic process	20	0.031
GO:0,042,180	Cellular ketone metabolic process	20	0.031
GO:0,044,271	Cellular nitrogen compound biosynthetic process	10	0.051
GO:0,006,418	tRNA aminoacylation for protein translation	5	0.057

#### Genotype effect on upregulation under drought conditions

Under conditions of water deficit, Tx7000 did not upregulate any significantly enriched biological process compared to SC56. On the other hand, SC56 upregulated a number of significantly enriched biological processes relative to Tx7000 (Additional File 2 and Table 5). Notably, the biosynthetic activity, especially that of proteins, was considerably higher in the drought-resistant, SC56. In addition to the heightened anabolic activity, transport as well as several metabolic processes were significantly enriched in S_treat > Tx_treat.

**Table 5:** Upregulated GO terms in SC56 compared to Tx7000 under drought

GO term	Term	# Genes	p value
GO:0,006,412	Translation	17	0.0022
GO:0,006,519	Cellular amino acid and derivative metabolic process	11	0.0031
GO:0,044,106	Cellular amine metabolic process	10	0.0054
GO:0,006,520	Cellular amino acid metabolic process	9	0.012
GO:0,034,660	ncRNA metabolic process	7	0.014
GO:0,009,308	Amine metabolic process	10	0.015
GO:0,055,085	Transmembrane transport	19	0.024
GO:0,006,399	tRNA metabolic process	5	0.052
GO:0,034,641	Cellular nitrogen compound metabolic process	10	0.053
GO:0,009,058	Biosynthetic process	55	0.054
GO:0,044,281	Small molecule metabolic process	19	0.058

## Discussion

Drought tolerance is a complex trait reflecting the intricacy of the abiotic stress response. When confronted with drought, plants alter the transcript abundance of a large number of genes with diverse functions and convoluted interactions. In roots, the drought cues are perceived by unknown sensors, and passed down through several signal transduction pathways, resulting in the expression of drought-responsive genes including those conferring tolerance. The convolution of drought response and adaptation is apparent in the present study as measured by the number of driven genes in SC56, which depicts a wider molecular response than Tx7000. Beyond drought response, the results suggest also expanded response of SC56 in water conditions.

### SC56 biologically outperforms Tx7000 in wet and drought conditions

In contrast to Tx7000_cont > SC56_cont, which showed no significantly enriched bioprocesses, SC56_cont > Tx7000_cont exhibited the highest count of enriched bioprocesses as well as DEGs. The mechanisms upregulated by SC56 compared to Tx7000 under wet conditions are all drivers of growth. Anabolism was the principal branch of metabolism that has been enhanced as indicated by more than hundred DEGs involved in the biosynthesis machinery with many contributing to translation of proteins as well as biogenesis of oligosaccharides. Also, metabolism related to amino acids (‘amino acids and derivatives,’ ‘oxoacid’ and ‘carboxylic acid’) was particularly enhanced in SC56; besides being the building blocks of protein, amino acids are involved in a plethora of cellular reactions influencing plant growth and development, generation of metabolic energy or redox power, and resistance to stress [11]. Furthermore, under optimal conditions, SC56 compared to Tx7000 exhibited unambiguous superior redox homeostasis (‘cell homeostasis,’ ‘cell redox homeostasis’ and ‘regulation of biological quality’) compared to Tx7000. In plants, ROS are continuously produced as a result of oxygen metabolism; if accumulated, ROS can lead to cell damage, but at certain levels have vital roles in cell signaling. Redox homeostasis is also crucial for proper cell functioning since various signaling pathways regulating cell division and stress reaction are sensitive to redox imbalance [12]. The above results point to the biological superiority of SC56 that possibly predisposes this genotype to mount an efficient response to drought. In particular, under wet conditions, ‘cell redox homeostasis’ was also overexpressed in another stay-green genotype (B35) [13]. This suggests redox balance at the pre-stress stage is an important feature of the stay-green character.

The outcomes of differential expression between SC56 and Tx7000 under drought also confirm a more efficient adaptation of SC56 to stress that is marked by better growth metabolism. While Tx7000_treat > SC56_treat showed no significantly enriched bioprocesses, SC56_treat > Tx7000_treat exhibited significantly enriched processes similar to those of SC56_cont > Tx7000_cont (‘biosynthesis,’ ‘translation,’ ‘amino acid and derivative metabolism,’ ‘ncRNA,’ ‘small molecule,’ and ‘tRNA’). Moreover, in conditions of drought, SC56 displayed enhanced ‘transmembrane transport’ with the overexpression of 19 proteins compared to Tx7000. This trait of the drought-resistant SC56 appears to be significant in the light of the increasing evidence that plant membrane transport systems play a substantial role in drought adaptation. Notably, the transmembrane transporter, Zinc-Induced Facilitator-Like 1 (ZIFL1) was overexpressed in SC56. A splicing isoform ZIFL1.3 was shown to mediate drought tolerance by regulating stomatal closure [14].

### SC56 overexpressed a plethora of stress tolerance genes in response to drought

To decipher the molecular basis of the drought resilience of SC56, gene expression of SC56 plants was compared to the drought-sensitive Tx7000 plants in dry conditions. As discussed, SC56_treat > Tx7000_treat exhibited a significantly larger number of DEGs than Tx7000_treat > SC56_treat. The examination of these DEGs relative to the known *Arabidopsis* drought-responsive genes uncovered that the ampler response of SC56 to water shortage also comprised a greater count of such genes since 57 were found in S_treat > Tx7000 versus only 18 in Tx_treat > S_treat. Relative to other stress resistances, resistance to drought is very challenging to evaluate since it is associated with a number of physiological, morphological, and molecular events. According to their function, drought-inducible genes can be classified into two groups. The first group encodes proteins that likely operate in stress tolerance and are referred to as ‘functional proteins’ while the second group referred to as ‘regulatory proteins’ [15] encodes factors involved in regulation of signal transduction and expression of genes putatively acting in stress response and are termed. With functional genomics advances, it has become evident that genes of both groups can confer stress tolerance.

#### Functional proteins

The functional proteins that were most frequently represented in our study are chiefly involved in enhancing the antioxidant capacity of SC56. Oxidative stress commonly occurs along with drought stress, causing lipid peroxidation, protein carbonylation, and DNA damage, which impairs their function and leads to deleterious effects on the cells. Plants have, thus, evolved a series of enzymatic and non-enzymatic antioxidant defense mechanisms to maintain the homeostasis of the intracellular redox state. In this study, there was a stronger antioxidant machinery in SC56 compared to the sensitive line Tx7000 under severe drought stress, as apparent by the overexpressed antioxidation-related genes. Glutathione *S*-transferases are a family of isozymes with the ability to catalyze the conjugation of the reduced form of glutathione (GSH) to xenobiotic substrates for the purpose of detoxification. In this study, GSTU18, GSTU7, GSTT1, GSTT3, GSTZ2, ERD9, DHAR2 (discussed latter) and AT1G65820 (microsomal GST) were all upregulated in SC56. Aside from GSTs, superoxide dismutases are a powerful antioxidant family involved in destroying superoxide free radicals. SOD1, which was upregulated in SC56, is a member of this family that was shown to enhance stress tolerance in plants [16]. Peroxidases are a class of proteins that breaks peroxides, and RCI3 is a member of this class that specializes in detoxifying hydrogen peroxide. RCI3, which was overexpressed in SC56, confers abiotic stress tolerance in plants [17, 18]. Tocopherols are lipophilic antioxidants synthesized exclusively by photosynthetic organisms and collectively constitute vitamin E. The enzyme VTE1, which was overexpressed in SC56 under drought stress is essential in the biosynthesis of tocopherols. In plants, tocopherols are synthesized in the chloroplasts where they protect membranes from oxidative degradation by ROS. VTE1 deficiency in *Arabidopsis* mutants leads to increased oxidative stress [19] whereas overexpression in tobacco prompts enhanced drought tolerance and increased chlorophyll levels [20]. Moreover, the simultaneous deficiency of VTE1 and GSH1 which is involved in glutathione biosynthesis results in oxidative stress that affects the stability and the efficiency of the photosynthetic apparatus [19]. In our study, glutathione metabolism and the biosynthesis of tocopherols were enhanced in SC56 under drought, hinting at better photosynthesis efficiency at least partly due to diminished oxidative stress.

The uncoupling proteins UCP1, UCP2, and UCP3 are a subgroup of the mitochondrial anion transporter family. The uncoupling of the mitochondrial electron transport chain from the phosphorylation of ADP optimizes the efficiency of oxidative phosphorylation and prevents generation of ROS by the respiratory chain. In plants, UCP1 is involved in maintaining the redox poise of the mitochondrial electron transport chain to facilitate photosynthetic metabolism. Disruption of UCP1 is associated with reduced photosynthetic carbon assimilation rate [21]. Furthermore, plants overexpressing UCP1 have better drought and salt tolerance and exhibit increased net photosynthesis, higher stomatal conductance, higher water retention and lower oxidative stress [22]. Thus, it appears that in conditions of abiotic stress, overexpression of UCP1 benefits the plant not only by alleviating the oxidative stress, but also by enhancing carbon assimilation. In the present study, UCP1 was upregulated in the drought-resistant SC56 genotype in conditions of water deficit.

Chloroplast-type ferredoxins (FDs) are electron transfer proteins that are involved in several metabolic processes including chlorophyll biosynthesis. FDs also participate in ROS scavenging by reducing the radical monodehydroascorbate to ascorbate. The ferredoxin isoforms FD1 and FD2, upregulated in SC56_treat > Tx7000_treat, are regulated by drought stress [23], and their knockout under heat stress was correlated to decreased ascorbate and adverse reactions to heat treatment, suggesting chloroplast FDs can confer stress tolerance [24].

Other functional proteins involved in drought tolerance in plants include proteinase inhibitors. Cystatin B (CYSB) is one such protein for which transcription was upregulated in SC56 compared to Tx7000 under drought treatment. CYSB overexpression in transgenic yeast and *Arabidopsis* plants increases the resistance to high salt, drought, oxidative, and cold stresses [25].

#### Regulatory proteins

In the present study, several previously reported drought-response regulatory genes (MAPK1, CRK7, CRK23, HVA22, CIPK1, CRK4, and CRK23) were upregulated in SC56 compared to Tx7000 under water deficit. Some of these genes have been validated as determinants of tolerance to drought and/or other stresses in plant systems. For instance, CBL1 and CBL9 perceive the Ca^2+^ signaling that is triggered by drought occurrence. Both factors, then specifically interact with CBL-interacting protein kinase 1 (CIPK1) to regulate the stress response which results into drought tolerance—loss of function of either gene results into sensitivity to drought [26]. Remarkably, CIPK1 represents a convergence point for abscisic acid (ABA)-dependent and ABA-independent stress response since CLB1 and CBL9 mediate both mechanisms, respectively. In plants, receptor-like protein kinases (RLKs), of which cysteine-rich receptor-like kinases (CRKs) are a subfamily, play essential roles in signal transduction by recognizing extracellular stimuli and activating the downstream signalling pathways. In *Arabidopsis*, the transgenic overexpression of different CRKs (CRK5, CRK4, and CRK19) resulted into the enhancement of ABA sensitivity and drought tolerance [27]. The member of this subfamily, CRK7, that was overexpressed in SC56, was also reported to be involved in stress tolerance through a protective role against apoplastic oxidative stress [28].

### SC56 overexpresses a negative regulator of senescence in response to drought

The stay-green trait reflects impaired or delayed chlorophyll catabolism and is divided into cosmetic stay-green, which is confined to pigment catabolism, and functional, in which the entire senescence syndrome, including chlorophyll catabolism, is delayed and/or slowed. The senescence syndrome is a complex set of processes characterized by the decline of photosynthetic activity, an overall metabolic switch from anabolism to catabolism, the degradation of macromolecules, and nutrient remobilization [29]. The initiation and progression of senescence can be inhibited by the potent senescence antagonists, cytokinins, and this route was used to create cytokinin-mediated stay-greens [30]. The senescence process can also be downregulated to produce stay-green phenotypes using mutated senescence-associated transcription factors [31]. In the present study, the gene encoding senescence-associated E3 ubiquitin ligase 1 (SAUL1, also known as PUB44), which is involved in chlorophyll biosynthesis and catabolism, was found to be more highly expressed in SC56 compared to Tx7000 under drought. In plants, SAUL1 negatively regulates premature senescence and cell death, as mutants lacking SAUL1 display early senescence [32]. Similarly, in a more recent study, PUB12 and PUB13 which encode U-box E3 ubiquitin ligases where found to negatively regulate stress-induced leaf senescence [33]. In sorghum, the complexity of the senescence syndrome is likely reflected in the molecular basis of functional stay-green since this phenotype is a classic example of a quantitative trait with continuous variation [34]. A drought study [9] involving a sorghum population derived from SC56 x Tx7000 uncovered a total of 9 quantitative-trait-loci (QTLs) in different environments, of which 3 (Stg A, Stg G, and Stg J) overlapped with QTLs uncovered in B35 [34, 35], the main source of stay-green in breeding programs. Thomas and Ougham [36] pointed out that the interactive nodes of transcriptional regulation, hormone- and ROS signaling, and sensors of environmental stresses that are associated with senescence offer massive number of junctures at which genetic modification can result in a stay-green phenotype, and constitute a rich source of variation for crop improvement. In this study, given the interconnectivity of drought tolerance networks and the high number of the obtained DEGs, SAUL1 might constitute one of several candidate genes that are significant for stay-green in SC56.

### SC56 overexpresses drought tolerance genes under wet conditions

The overexpression of drought response genes in wet conditions before the onset of drought stress might predispose plants for a more efficient response to stress, which might be the case of the stay-green phenotype, since it was also observed in wet conditions [37]. SC56_cont > Tx7000_cont showed more than fifty *Arabidopsis* known drought-responsive genes with several shown to confer stress tolerance by transgenic overexpression or by knockdown (in *Arabidopsis* or tobacco), as discussed hereafter. Trehalose-6-phosphate synthase (TPS1), which is critical for the biosynthesis of the osmoprotectant trehalose was linked to dehydration tolerance [38]. Interestingly, TPS1 was also overexpressed under wet conditions in a study comparing sorghum stay-green line B35 to the senescent line R16 [13]. Similarly, the UB-like protease 1D (ULP1D) confers tolerance to different stresses [39, 40]. This protein is a deSUMOylating enzyme, which in plants is associated with developmental mechanisms and stress responses through the post-translational regulatory process of SUMOylation/deSUMOylation. Pyrophosphorylase 6 (PPa6), shown to be involved in drought tolerance [41], is part of a group of enzymes that catalyses the hydrolysis of PPi to Pi, which is central to many anabolic processes.

In SC56_cont > Tx7000_cont, many of the upregulated known stress tolerance genes are associated with protection against oxidative stress: the antioxidants superoxide dismutases SOD1 and SOD2 [42], vitamin C defective 1 (VTC1) [43], glyceraldehyde-3-phosphate dehydrogenase C subunit 1 (GAPC1) [44], monodehydroascorbate reductase 1 (MDAR1) [45], methionine sulfoxide reductase B 2 (MSRB2) [46], and ferritin 2 [47]. In relation to the enhanced growth capacity of SC56, possibly MSRB2 is especially relevant since, in *Arabidopsis*, plastidial MSRB1 and MSRB2 account for most leaf peptide MSR activity and have been shown to be essential for growth under environmental constraints due to their involvement in the preservation of the photosystem antennae [46]. In the context of the stay-green phenotype, ABC1-like kinase 1 (ABC1K1), which is upregulated in SC56 compared to Tx7000 under wet conditions, might be of particular interest since it is involved in modulating chlorophyll degradation directly by maintaining the number of Chl-binding photosynthetic thylakoid membranes and by playing a role against photooxidative stress [48]. The overexpression of numerous stress tolerance genes in non-stress conditions suggests that the transcriptional makeup of SC56 prior to the onset of stress might contribute to a strong and early stress response potentially involving a network of genes that culminate into high stress tolerance. However, despite the stress tolerance role of the abovementioned genes and despite their overexpression in SC56 under wet conditions, it is difficult to corroborate this role in this study since there was no differential upregulation in the drought treatment. Nevertheless, these genes might have been differentially expressed during an earlier time point of the drought stress that was not captured during the late drought response in these experiments (transcription levels measurement at 13 days post-drought, with an SMC of less than 10%).

Some genes that are possibly highly associated with drought resilience of SC56 would be those overexpressed under both wet and dry conditions compared to Tx7000. The scrutiny of the common DEGs in the relevant comparisons (S_cont > Tx_cont and S_treat > Tx_treat) revealed several stress response genes including glutathione transferases and heat-shock proteins. Most notably, copper/zinc superoxide dismutase 1 (SOD1), CBL-interacting protein kinase 1(CIPK1) and dehydroascorbate reductase 2 (DHAR2) were overexpressed in SC56 under both conditions and have been found to play a role in stress tolerance. As mentioned, SOD1 is a crucial ROS scavenging enzyme, and was shown to enhance oxidative stress tolerance via transgenic overexpression in tobacco [16] and in *Arabidopsis* [49]. Similarly, as discussed, CIPK1 is a regulatory protein that is a convergent point in ABA-dependent and ABA independent stress tolerance [27]. CIPK1 was also overexpressed in the stay-green line B35 compared to the senescent line R16 under wet conditions [13]. DHAR2 is a dehydroascorbate reductase (DHAR), which by reducing the oxidized form of ascorbic acid regulates its cellular redox state, and thus affects cell responsiveness and tolerance to environmental ROS [50]. The overexpression of DHAR2 in SC56 in comparison to Tx7000 is possibly a key element in the difference of drought tolerance between both lines. In a study using transgenic tobacco [50], suppression of DHAR caused a preferential loss of chlorophyll a, lower levels of the carbon fixing enzyme Rubisco, and a lower rate of CO_2_ assimilation that correlated with a slower growth and reduced foliar dry weight. In addition, premature leaf aging was observed in mature leaves as seen through an accelerated rate of loss of chlorophyll, Rubisco, light-harvesting complex II, and photosynthetic functioning. Conversely, DHAR overexpression sustained higher levels of chlorophyll, rubisco, light-harvesting complex II, and photosynthetic functioning while maintaining lower levels of lipid peroxidation, resulting in delayed leaf aging. Hence, by recycling ascorbic acid, DHAR possibly protects against ROS-mediated damage and affects the level of photosynthetic activity, thus influencing the rate of plant growth and leaf aging.

## Conclusion

This study uncovered differences in the molecular determinism of SC56 and Tx7000 under drought as well as under water conditions. Under irrigation, SC56 compared to Tx7000 upregulated mechanisms driving growth and ensuring homeostasis. The biological capacity of SC56 to withstand drought spanned constitutive attributes, principally acting in defense against oxidative stress. The potency of the antioxidant machinery of SC56 in attenuating water stress comes into play outwardly in the drought treatment through overexpressed genes known as stress tolerance determinants active in combatting ROS-mediated damage. In addition, SC56 drought response was centered on the upregulation of transmembrane transport capacity, making it possible to uphold anabolic and metabolic processes (translation, small molecule metabolism, etc.) under environmental stress. This study, thus, correlates the drought tolerance of SC56 to the overexpression of bioprocesses supporting a better accumulation of biomass in optimal as well as limiting water conditions. This study also highlights that SC56 genotype overexpresses a plethora of genes that confer stress tolerance.

## Electronic supplementary material

Below is the link to the electronic supplementary material.

Additional file 1: Table S1. List of drought-responsive genes of *Arabidopsis thaliana*. The genes were identified using the database, Phytozome (https://phytozome.jgi.doe.gov/pz/portal.html). The ‘keyword search’ in ‘Tools’ was utilized. The keywords used for the search were: drought, tolerance, stress, and adaptation. Table S2. Overlap of the DEGs list by experimental group and the *Arabidopsis* drought-responsive gene list. The DEG lists issued from the differential expression analysis of S_treat vs. Tx_treat, S_cont vs. Tx_cont, S_treat vs. S_cont, and Tx_treat vs. Tx_cont were compared individually to the *Arabidopsis* drought-responsive gene list to determine overlap. The resulting genes constituted the known drought-responsive genes of the respective experimental group. Table S3. Combined overlap of the DEGs lists and the *Arabidopsis* drought-responsive gene list. The DEG lists issued from the differential expression analysis of S_treat vs. Tx_treat, S_cont vs. Tx_cont, S_treat vs. S_cont, and Tx_treat vs. Tx_cont were compared to the Arabidopsis gene list and the resulting overlap was combined in a list that constitutes the drought-responsive DEGs of the present study. Supplementary file 1 (XLSX 61 kb)

Additional file 2: **Table S1**. Significantly enriched GO terms in SC56 drought treatment compared with SC56 control. **Table S2**. Significantly enriched GO terms in Tx7000 drought treatment compared with Tx7000 control. **Table S3**. Significantly enriched GO terms in SC56 control compared to Tx7000 control. **Table S4**. Significantly enriched GO terms in SC56 drought treatment compared to Tx7000 drought treatment.. Supplementary file 2 (XLSX 85 kb)

## Data Availability

The raw data of the RNA-Seq datasets was deposited in Sequence Read Archive (SRA) under the Submission Number PRJNA556240.

## Abbreviations

ABA: Abscisic acid
ABC1K1: ABC1-like kinase 1
CBL1: Calcineurin B-like protein 1
CBL9: Calcineurin B-like protein 9
CIPK1: CBL-interacting protein kinase 1
CRKs: Cysteine-rich receptor-like kinases
CRK4: Cysteine-rich receptor-like kinase 4
CRK5: Cysteine-rich receptor-like kinases 5
CRK7: Cysteine-rich receptor-like kinase 7
CRK19: Cysteine-rich receptor-like kinase 19
CRK23: Cysteine-rich receptor-like kinase 23
CYSB: Cystatin B
DEGs: Differentially expressed genes
DHAR2: Dehydroascorbate reductase 2
ERD9: Early-responsive to dehydration 9
FD: Ferredoxins
GAPC1: Glyceraldehyde-3-phosphate dehydrogenase C subunit 1
GO: Gene ontology;
GSH1: Glutamate-cysteine ligase
GSTs: Glutathione *S*-transferases
GSTT1: Glutathione *S*-transferase theta 1
GSTT3: Glutathione *S*-transferase theta 3
GSTU18: Glutathione *S*-transferase TAU 18
GSTU7: Glutathione *S*-transferase TAU 7
GSTZ2: Glutathione *S*-transferase (class zeta) 2
HVA22: Abscisic acid-responsive (TB2/DP1, HVA22) family protein
MAPK1: Mitogen-activated protein kinase 1
MDAR1: Monodehydroascorbate reductase 1
MSRB1: Methionine sulfoxide reductase B 1
MSRB2: Methionine sulfoxide reductase B 2
NGS: Next-generation sequencing
Pi: Inorganic phosphate
PPa6: Pyrophosphorylase 6
PPi: Inorganic pyrophosphate
PUB12: Plant U-box (PUB) E3 ubiquitin ligase 12
PUB13: Plant U-box (PUB) E3 ubiquitin ligase
RCI3: Rare cold inducible gene 3
RLKs: Receptor-like protein kinases
RNA-Seq: RNA sequencing
ROS: Reactive oxygen species
RPKM: Fragments Per Kilobase of transcript per Million fragments mapped
SAUL1: Senescence-Associated E3 Ubiquitin Ligase 1
SMC: Soil moisture content
SOD1: Superoxide dismutase
Stg A: Stay-green A
Stg G: Stay-green G
Stg J: Stay-green J
TPS1: Trehalose-6-phosphate synthase
UCP1: Plant uncoupling mitochondrial protein 1
UCP2: Plant uncoupling mitochondrial protein 2
UCP3: Plant uncoupling mitochondrial protein 3
ULP1D: UB-like protease 1D
VTC1: Vitamin C Defective 1
VTE1: Vitamin E deficient 1
ZIFL1: Zinc-Induced Facilitator-Like 1
